# Breakage of Tapered Junctions of Modular Stems in Revision Total Hip Arthroplasty—High Incidence in a Consecutive Series of a Single Institution

**DOI:** 10.3390/bioengineering10030341

**Published:** 2023-03-08

**Authors:** Oliver E. Bischel, Arnold J. Suda, Paul M. Böhm, Therese Bormann, Sebastian Jäger, Jörn B. Seeger

**Affiliations:** 1 Berufsgenossenschaftliche Unfallklinik Ludwigshafen, Ludwig-Guttmann-Str. 13, 67071 Ludwigshafen, Germany; 2 AUVA-Unfallkrankenhaus Salzburg, Dr.-Franz-Rehrl-Platz 5, 5010 Salzburg, Austria; 3 General Orthopedics, Neumeyerstr. 46, 90411 Nuremberg, Germany; 4 Department of Orthopedics and Traumatology, University of Heidelberg, Schlierbacher Landstr. 200a, 69118 Heidelberg, Germany; 5 Kurparkklinik, Kurstr. 41-45, 61231 Bad Nauheim, Germany

**Keywords:** modular revision stem, revision THA, breakage of taper junction, survivorship analysis

## Abstract

Background: Modularity in revision THA (RTHA) has become accepted during the last three decades. Nevertheless, specific risks of modularity of current revision devices such as breakage of taper junctions occur during follow-up. Data reporting failure rates are predominantly given by the manufacturers but independent data acquisition is missing so far. Questions/Purposes: 1. What time-related risk of breakage of taper junction between neck and body of an established modular revision device can be expected in a consecutive single institutional series and a mid-term follow-up? 2. Are there specific factors influencing breakage in this cohort? Materials and Methods: A retrospective analysis was performed of a consecutive series of 89 cases after femoral revision using a tapered modular revision stem. Mean follow-up period was 7.1 (range: 3.0–13.7) years. Breakage of stem as failure criteria of the implant was investigated with a Kaplan–Meier analysis. Results: Breakage of taper junctions occurred in four patients during follow-up showing a time-depending implant survival of 94.2 (95% CI: 88.6–100%) after 13.7 years. Implant survival of stems with lateralized necks of 87.4 (95% CI: 75.6–100%) after 13.7 years was significantly lower compared to the standard offset variant with 100% after 13.5 years (log rank test *p* = 0.0283). Chi square test also revealed a significantly higher risk of breakage of lateralized necks compared to standard offset pieces (*p* = 0.0141). Three of four patients were obese with a mean BMI of 37.9 kg/m^2^. Grade of obesity (grade 1 or higher) had significant influence on risk of breakage. Survival of the implant was significantly lower in obese patients with at least grade 1 obesity compared to patients with a BMI < 30 kg/m^2^ (82.9 (95% CI: 64.9–100%) after 11.6 years vs. 98.4 (95% CI: 95.3–100%) after 13.7 years; log-rank *p* = 0.0327). Conclusions: Cumulative risk for failure of taper junctions was high in this consecutive single institutional cohort and may further increase during follow-up. As independent data acquisition in registries is missing, failure rate may be higher than reported data of the manufacturers. The use of lateralized offset necks in obese patients of at least grade 1 obesity showed a significantly higher risk of breakage. The use of monobloc revision devices may be an option, but randomized control trials are currently missing to establish standardized treatment protocols considering individual risks for both monobloc and/or modular implants.

## 1. Introduction

### 1.1. Background

According to current registry data, more than 10% of all hip arthroplasty procedures had been revisions (German Arthroplasty Registry, Annual report 2021; https://www.eprd.de/en/downloads/reports, accessed on 17 January 2023). In addition, exchange of the stem occurred in nearly 50% of all RTHA procedures. The use of tapered monobloc revision stems has shown to be reliable and safe with excellent mid to long-term results and conical designs may be advantageous compared to cylindrical monobloc implants [1,2,3]. Modular revision stems were introduced in RTHA approximately three decades ago and most currently available devices are combining both an approved tapered stem design and a modular built up. Survival rates of 94% of modular devices with revision due to any cause as the end point have been published in short- to medium-term studies [4,5]. Modularity offers a certain reliability and flexibility to the surgeon. Insertion of the stem can be performed more easily without compromising primary stability. Reconstruction of patients’ hip biomechanics or addressing problems such as dislocation due to missing length or femoral head offset can be adapted intraoperatively or during follow-up without withdrawing the complete implant. Nevertheless, adverse effects such as corrosion, fretting, debris formation, disconnection and even breakage of the stem may appear [6,7,8].

### 1.2. Rationale

There are few data available of some manufacturers giving a mechanical failure rate of the junction of estimated 0.30% [9]. Of the few data available from independent sources, such as current registries, revision due to implant fracture after former revision is 2.8% (National Joint Registry for England, Wales, Northern Ireland and the Isle of Man, 16th Annual Report, 2019; http://reports.njrcentre.org.uk/2018, accessed on 17 January 2023), including all sorts of breakage including taper junctions. Consecutively, it is unclear what incidence of breakage has to be estimated in correlation to potential other failure reasons like infection, periprosthetic fracture or aseptic loosening in a mid- or long-term duration. Specific influences on breakage of taper junctions such as obesity, type of defect, offset or length of the neck segment have been shown in case reports, retrospective or in vitro investigations of modular implants [10,11,12].

The following questions were posed. What time-related risk of breakage of taper junction of an established modular revision device can be expected in a consecutive series and a mid-term follow-up? Are there any influencing factors for breakage of the taper junctions?

## 2. Materials and Methods

**Figure 1:**
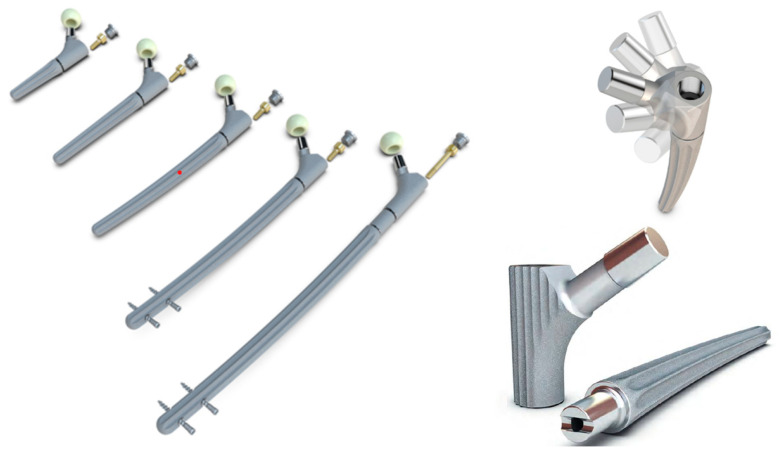
Implant characteristics MRP^®^ revision stem: straight stems of 80, 140 and 200 mm length are available and curved stems of 200, 260 and 320 mm length. Three neck lengths (50, 60 and 70 mm) and a lengthening piece between distal stem and neck of 30 mm allows a continuous adaptation of the implant length between 130 and 420 mm in 10 mm steps. A diameter beginning from 13 up to 25 mm is available for the 80 mm stem and between 13 to 30 mm for the 140 and 200 mm device. The range of the diameter is from 11 to 29 mm for the 260 and 320 mm option. There are two neck options connectable, a standard offset neck and a lateralized piece with an increased offset of 10 mm.

A consecutive series of 130 revisions between 2003 and 2009 performed with the MRP^®^ revision stem (Peter Brehm, Weissendorf, Germany) was evaluated retrospectively (Figure 1). A follow-up of more than three years as inclusion criteria for this study showed 89 patients (47 women and 42 men; 41 right and 48 left hip joints). Mean age at surgery was 66 (range: 37–85) years.

Indication for surgery was aseptic loosening in 44 cases and periprosthetic fracture in 10 hip joints. A total of 35 septic cases required two-stage revisions. All patients were evaluated clinically and radiologically. Defect classification was carried out according to the classification of Paprosky [13]. Body mass index (BMI) was used to determine obesity. Statistic calculations were performed after division into the following groups: underweight, normal weight or preobesity vs. grade 1 or higher obesity (>30 kg/m^2^); underweight, normal weight, preobesity and obesity of grade 1 and 2 vs. obesity of grade 3 (>40 kg/m^2^).

An ethical vote of the institutional ethics committee was obtained (S-096/2012). All patients had signed a consent form and anonymized data were used for evaluation. Statistical analysis was performed with JMP 10 for Mac (SAS Institute Inc., Cary, NC, USA). A time to event analysis was performed using the Kaplan–Meier method with removal of the stem for any cause, aseptic loosening and/or breakage of the stem, and worst case (removal of the stem for any cause and/or aseptic loosening and/or lost to follow-up) serving as failure criteria. A 95% confidence interval was given to all survivorship data; the *p*-value for comparing survival curves was calculated with the log-rank-test. Associations or correlations between a continuous and/or discrete variable were tested by Student’s *t*-test, Paired *t*-test or Chi square test, depending on the underlying empirical distribution. All tests were two-sided and *p* ≤ 0.05 was considered significant. The data were evaluated descriptively using the arithmetic mean, SD, range and 95% confidence intervals.

### Surgical Technique

An anterolateral approach was used in 72 cases. Revision by a transfemoral approach after anterolateral joint exposure was performed in 17 hips. Allograft bone was used during operation at the femur for defect reconstruction of the tube in 17 cases. Strut-grafts with additional morselized allo- and/or autograft gained from the cup during reaming were implanted in four cases and morselized material only in 13.

The MRP^®^ revision system was used as a cementless device in all cases. A press-fit situation was approached for primary stability. All straight 140 mm (n = 26) and 200 mm (n = 3) stems were implanted after preparation of the femoral canal with rasps. All longer distal anchoring devices were used as curved stems (200 mm, n = 53; 260 mm, n = 6; 320 mm, n = 1) and preparation was performed with flexible reamers.

## 3. Results

Mean follow-up period was 7.1 (range: 3.0–13.7) years and data were available for all revisions (n = 89). Five patients died after a mean duration of 7.2 (range: 3.3–11.6) years postoperatively. Data of these patients were included until their last follow-up.

### 3.1. Complications

There were seven failures during follow-up, four due to breakage of taper junction and three infections. Preserving therapy was successful in another three infected hips. There were seven dislocations during follow-up and there was no influence of the used neck on the dislocation rate (standard neck n = 4; lateralized n = 3). Four were treated by open reduction and exchange of the cup. Another three cups were revised during follow-up due to aseptic loosening. One of them had a second cup revision after repeated loosening.

### 3.2. Risk Factors for Breakage of the Taper Junction

Fracture of the taper junction occurred in four arthroplasties after a mean period after surgery of 4.3 (range: 2.8–5.5) years (Table 1). Experience of the surgeon showed no significant influence although two of the four failures occurred with one surgeon within the first seven revisions with this system. All but one case showed overweight with a mean of 37.9 (27.1, 32.7, 44.3 and 47.5) kg/m^2^. Obesity of grade 3 showed a significantly higher correlation of breakage (two out of four vs. two out of 85; Chi square test *p* = 0.0043). In all four cases, no lengthening piece but lateralized necks were used. Chi square test revealed significant higher risk with use of lateralized (n = 43) compared to standard necks (n = 46) (*p* = 0.0141). The type of postoperative defect according to the classification of Paprosky is given in Table 2. Postoperative defect showed no influence on stem breakage. At time of breakage all four patients showed a type 2 defect due to at least partial restoration according to Paprosky, but this was also not of significant influence on stem breakage during follow-up.

### 3.3. Survival Analysis

**Figure 2:**
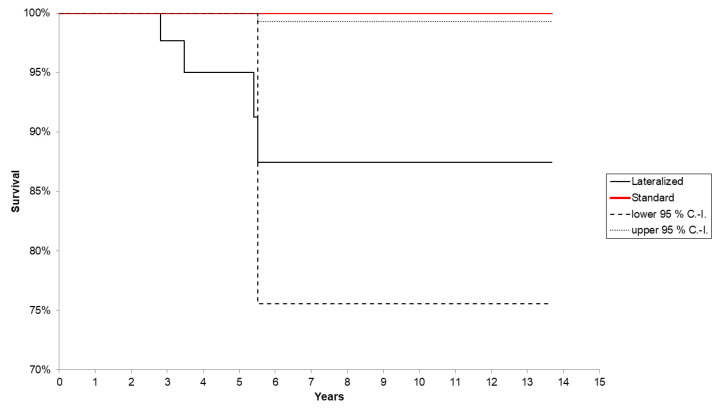
Implant survival with breakage of taper junction as endpoint-lateralized vs. standard offset (log rank *p* = 0.0283; 95% confidence interval).

Implant survival with stem breakage as the endpoint was 94.2 (95% CI: 89.6–100%) at 13.7 years. Survival of the implant using a lateralized neck was significantly lower compared to systems built up with standard necks (Figure 2 and Table 3). BMI also had an influence on implant survival with breakage of the taper junction as the endpoint (Table 3).

Experience of the surgeon, defect classification, defect regeneration or length or diameter of the distal anchoring piece had no influence on the risk of breakage of the stem.

### 3.4. Retrieval Analysis of Coupling

**Figure 3:**
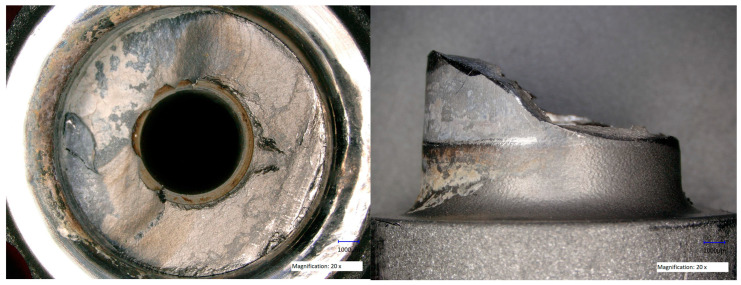
Neck piece (**left**) and lateral view of the broken taper (**right**).

**Figure 4:**
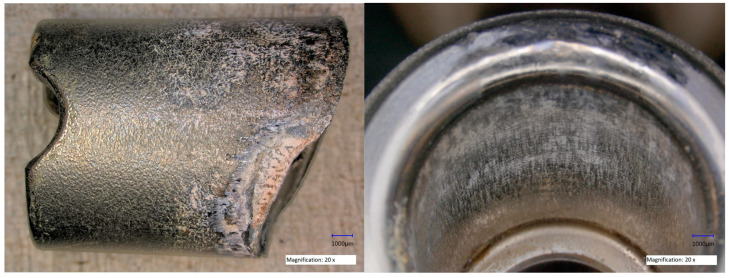
Neck (**left**) with broken proximal taper piece after disconnection (**right**).

**Figure 5:**
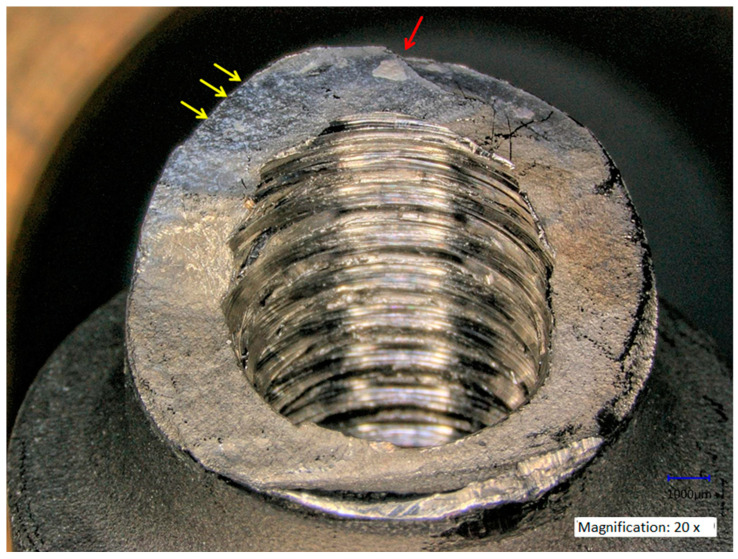
Broken taper with screw thread for extraction-lateral initiation of the crack with progression in medial direction (red arrow in the region with bright deposits marks the area of crack initiation; occurring rest lines are marked with yellow arrows).

**Figure 6:**
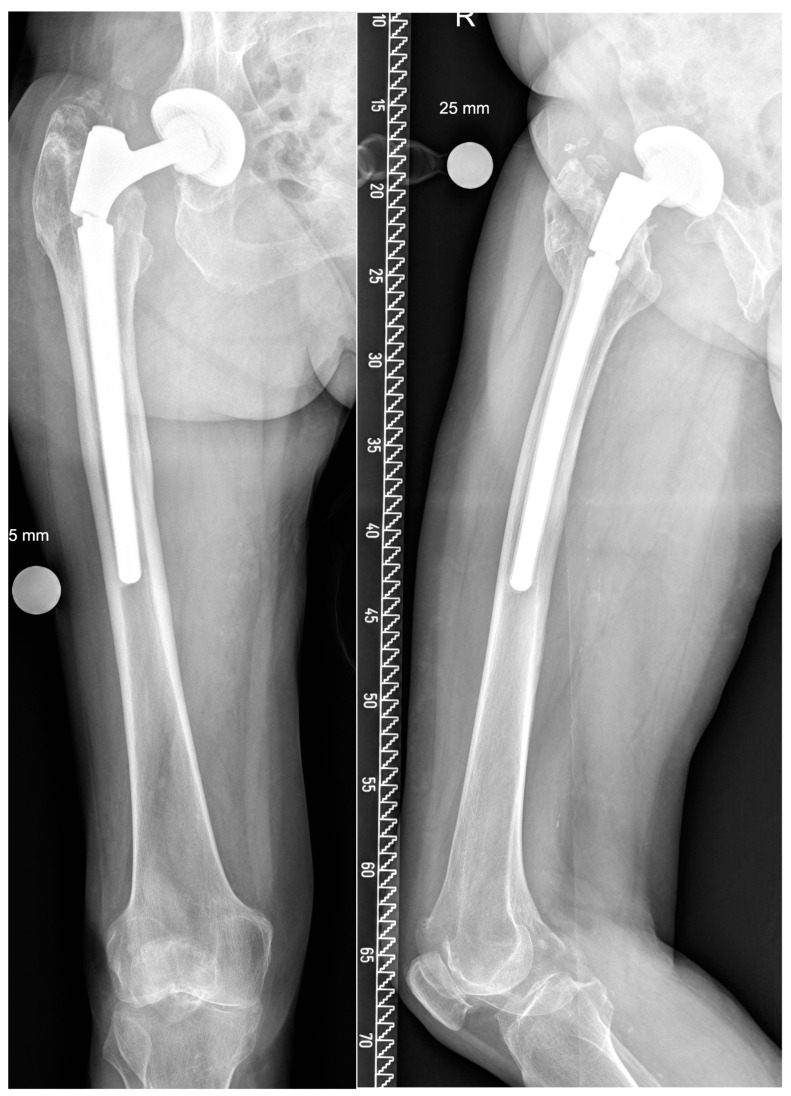
Patient (47 years, male) with minor bone defect and distally integrated stem until junction area.

**Figure 7:**
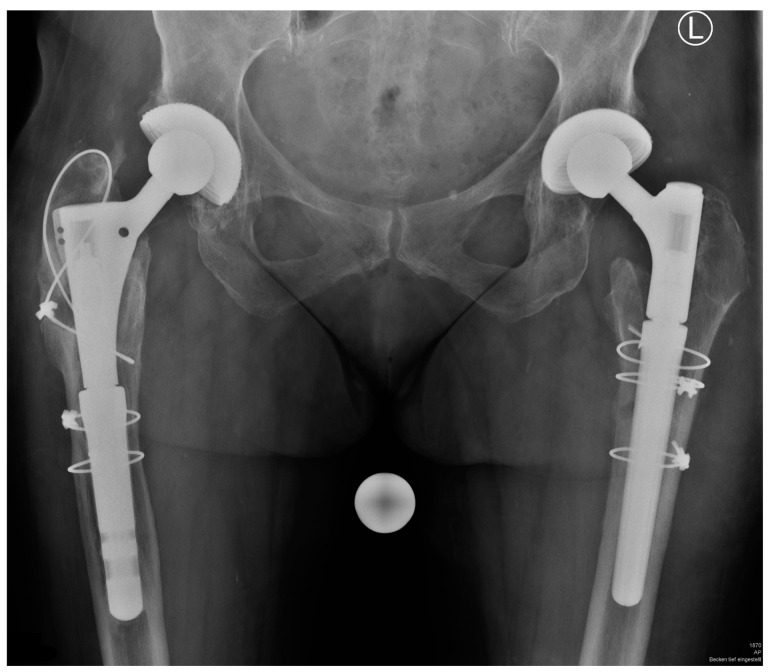
Patient T. R. after 4 years postoperatively by transfemoral approach. Revision was performed by another modular tapered revision device. A shorter system with another tapered modular revision system was used for the type 2 defect. The junction area is more distal with a supportive bone situation at the neck proximal to the junction. Meanwhile, a periprosthetic fracture occurred on the left side with identic therapy in a type 2 defect (1 year postoperatively).

Two of the four explants with breakage of the taper junction could be examined in vitro. The two explants showed identic failure patterns. Disconnection of the fractured proximal piece of one stem was performed by a universal material testing machine (MTS 858 Mini Bionix, MTS Systems, Eden Prairie, MN, USA). Traverse speed was 0.008 mm/sec. The force to disconnect the broken stem piece out of the neck was 15.06 kN. Photographs were made before (Figure 3) and after disconnection (Figure 4; twentyfold optical enlargement). Figure 5 shows the distal part of the broken taper after disconnection. The crack originated laterally and progressed in the medial direction, which is evident from the bright deposits (red arrow) in the area of crack initiation as well as occurring rest lines (yellow arrows). Residual fracture caused by overload is located medially, opposite from the crack origin. X-rays of the patient before and after revision of the broken device are given in Figure 6 and Figure 7.

## 4. Discussion

### 4.1. Background and Rationale

The use of modular endoprostheses has become popular in RTHA and after tumor resection for reconstruction of bone defects. Since its introduction nearly three decades ago, only a few studies have been published with a large cohort of patients and at least a mid-term follow-up duration [3,4,5,14,15,16,17].

One of the advantages of modularity may be the reconstruction of patients’ biomechanics with femoral head offset and leg length. In addition, an intraoperative or postoperative adaptation with exchange of the proximal components may be performed easily, while the well-fixed distal anchoring part can be left in situ.

### 4.2. Cumulative Risk of Stem Breakage

Nevertheless, wear, fretting, cold welding and corrosion of the taper junctions of modular devices are described and have to be taken into account as recent publications have shown [6,18,19,20,21]. The risk for breakage due to the mentioned reasons may increase over the years, as it is a creeping phenomenon. This finding is also derivable from current studies dealing with identic modular devices, showing a decreasing survival through the years due to stem breakage [22]. There were four mechanical failures in this cohort by means of breakage of taper junctions between stem and neck after a mean of 4.3 (range: 2.8–5.5) years after implantation.

Compared to the available data, an absolute rate of 4.5% (4 out of 89) is higher than the described risks of 1.4% (1 out of 70) or 3.6% (6 out of 165) of comparable modular devices [11,23] and is not reported with the same implant in a comparable study [24]. There is one recent publication of the manufacturer of this implant, giving an absolute rate of mechanical failure by means of junction fracture of 0.3% (113/37600) [9]. In the annual report of 2019 of the National Joint Register (NJR), stem breakage of 2.8% as an indication for rerevision of the stem is published (National Joint Registry for England, Wales, Northern Ireland and the Isle of Man, 16th Annual Report, 2019). As a detailed analysis is not available, it remains unclear whether a monobloc or modular device cracked or in the latter the body or taper junctions failed. Nevertheless, an absolute number of 4.5% in this study suggests a higher rate of junction cracks than reported even of the investigated system.

Cumulative, and therefore, time-dependent risk of stem fracture may be more meaningful, as absolute numbers to estimate this mechanical problem but are not given in the current literature. A cumulative risk of 5.8% after 13.7 years in this cohort is approximately as high as the failure risks for aseptic loosening or infection and should, therefore, cause major concerns to surgeons dealing with RTHA. Due to our data, central and independent registration of specific mechanical failure reasons is mandatory to estimate individual risks of each implant dependent on patients’ characteristics such as bone defect, BMI or offset variant. Additionally, correlation between other failure reasons, such as infection or aseptic loosening, may be possible. Finally, estimation of implant-specific risks must be known to develop an algorithm in patients undergoing femoral RTHA.

### 4.3. Lateralized Neck and Obesity As a Risk Factor

Modularity and especially the possibility of different offset options have been proposed to have positive influence on functional outcome and complications such as dislocation. A dislocation rate of nearly 8% (7/89) in this cohort corresponds with the rate of current literature of between 9 and 10% [25,26]. In addition, the dislocation rate was not influenced by the used offset variant. We agree with Regis et al. that the use of modular necks with different offset variants alone may not lower the dislocation rate [27]. Adaption of leg length and offset in primary THA does not necessarily result in better functional results. We have observed a significantly higher risk of breakage of the taper junction in stems built up by a lateral offset neck (n = 4) compared to a standard variant (n = 0). Obesity of at least grade 1 had also had a significant influence on risk of breakage increasing with the grade of obesity (grade 1:3 out of 26 vs. 1 out of 63; grade 3:2 out of 4 vs. 2 out of 85). Due to these findings, the decision for a lateralized neck should be made carefully in obese patients of at least grade 1 (>30 kg/m^2^). Although not of significant influence, a defect of type 2 or 3 A according to Paprosky at time of operation was present in all fractured cases. This may be another factor to be considered when dealing with modular systems in RTHA. Factors such as a dislocation rate and risk of revision due to implant related complications in addition to overall implant survival may lead to the decision pro or contra modularity with use of a standard or lateralized offset [28].

### 4.4. Further Factors Influencing Risk of Breakage and Technical Considerations

Furthermore, a possible failure reason may be repeated connection of taper junctions as performed in one patient in this study (Table 1). This may be necessarily performed in patients presenting with instability to lengthen the system and/or adapt offset by exchanging the neck and/or inserting a lengthening piece. Especially in infections treated with a preservative regimen, e.g., vacuum therapy with instillation, risk for failure may increase as a couple of revisions with disconnection of the mobile parts of the system at any time are usually performed to clean the components mechanically until definitive restoration of the system by new parts at latest revision. Postoperatively, two patients showed a type 3A situation with a partial bone restoration during follow-up, leading to a type 2 defect according to Paprosky in all four cases at time of breakage. As bone restoration occurred within a couple of months, type 2 defects were present for a longer period of time in all four cases. Minor defects, such as a type 2 defect with rigid and osseointegrated fixation of the distal stem component and a defect situation beginning near the junction area up to the neck more proximally, may give a maximum mechanical stress to the junction area leading to fatigue stress after initial repeated bending. The two explants of this cohort showed identic failure patterns. The disconnection force exceeded 15 kN. Due to this finding and as other obvious failure reasons are missing, a technical error during implantation is unlikely, as the taper connection was tight. Repeated bending leading to fatigue stress at the junction may be the main reason for failure, although signs of corrosion were present. This is in accordance with the findings of Lakstein et al. [11] or Fink [10].

Due to these findings, an operative technique when using modular systems should be carefully adapted and a situation described above should be avoided. The use of longer necks leading to a more distal location of the junction with better bony support around it as well as additional bone grafting is proposed [12]. Nevertheless, a bony restoration is difficult to achieve especially after transfemoral approach or extended trochanteric osteotomy with highly deficient bony lids at the proximal femur. The use of (massive) allografts may also leave some space between bone and neck with persisting instability due to delayed or only at least partial ingrowth.

A greater diameter with massive junction body and longer connection length may be the reason for a potentially lower risk of breakage in tumor devices. In contrast, the diameter of the junction is generally smaller and the length has been shortened during time at different revision devices [19]. Therefore, adaptation of length and diameter of the taper junction and/or a hardening of the material may also be an option but should be investigated in further studies [11,29].

A lateralized offset combined with obesity of at least grade 1 and a type 2 or type 3A defect at time of implantation (decreasing to a type 2 defect at breakage due to defect regeneration) are the main risk factors in this cohort. The use of monobloc devices especially in cases with limited bone defect of type 1 or 2 according to Paprosky may be an option and implants like the Wagner SL^®^ (Zimmer Biomet, Warsaw, IN, USA) revision device may get certain a renaissance [2,30,31]. Nevertheless, breakage may also occur with monobloc devices with ingrown stems at the isthmus and a defect situation more proximally due to the same biomechanical reasons. As a potential increase may occur during follow-up due to mechanical stress for both monobloc and modular devices, further long-term data and research is necessary to assess the pros and cons of modularity in relation to monobloc devices within the context of defect reconstruction in revision hip or tumor arthroplasty. To prevent junction breakage, especially the technical challenges mentioned above have to be examined in detail and opposed to monobloc options, which has existing challenges due to its straight design. In the case of a broken stem element or revision of a well-integrated, distally fixed stem, exchange is challenging and even impossible without a transfemoral approach and further potential excessive bone damage.

### 4.5. Limitations

The included number of patients is relatively low and the follow-up period is limited. Nevertheless, a consecutive series with 89 patients in a medium-term follow-up seems to be sufficient to answer the questions asked. As comparable studies with a relevant number of patients are missing, it remains unclear what cumulative fracture risk of other modular devices can be expected. We could not detect a negative selection of patients in this cohort.

## 5. Conclusions

Although intra- and postoperative adaption to patient’s characteristics is easier to perform with a modular device, breakage of taper junctions appears and the risk may rise during follow-up. The use of lateralized offset necks of this implant in patients with an at least grade 1 obesity shows a significantly higher risk of breakage compared to the standard offset variant. There is a higher rate of junction cracks in this consecutive single institutional series than previously reported by the manufacturer of this system. Due to the lack of independent data sources such as registries or even long-term follow-up studies, the exact number of junction failures of used modular systems is difficult to estimate. Although technically more demanding, monobloc devices may be an option in patients that are at risk (type 2 defect, obesity and high offset).

## Figures and Tables

**Table 1:** Breakage of taper junction.

Patients Sex, Age (Year)	Indication ^#^	Stem Configuration *	BMI	Bone Defect	Breakage Po. (Year)
m., 82	ppf	c, 21, 200, lat, long, m	27	3A	2.8
f., 55	al	s, 16, 140, lat, short, s	33	2	5.4
m., 58 ^§^	al	s, 30, 200, lat, short, s	44	3A	3.5
m., 47	ts	c, 16, 200, lat, short, s	45	2	5.5

^#^ periprosthetic fracture (ppf), aseptic loosening (al), two stage septic revision (ts). * curved © or straight (s), diameter (mm), length (mm), neck lateralized (lat) or standard (std), length of neck (long, medium, short), length of head (s = short, m = medium, l = large, xl = extra large). ^§^ The patient had acromegalogigantism (196 cm, 170 kg) and breakage of the junction occurred 3.1 years after successful THA-preserving treatment of an infection. For best treatment of infection by vacuum therapy with instillation, all mobile parts (head, neck and inlay of the cup) had been removed for mechanical cleaning five times during the revisions until the definitive sixth exchange with implantation of new mobile parts at the latest revision (neck, head and inlay of the cup).

**Table 2:** Defect situation.

**Paprosky**	2	3A	3B	4
**No. of Cases**	32	18	31	8

**Table 3:** Survivorship analysis.

Group	Survival
Overall	94.2 (95% CI: 88.6–100%) after 13.7 years
Lateralized (L) vs. standard neck (S) (log rank test *p* = 0.0283)	L: 87.4 (95% CI: 75.6–100%) after 13.7 years (n = 4) S: 100% after 13.5 years
BMI: overweight > 30 kg/m^2^ (O) vs. normal weight (N) (log rank test *p* = 0.0327)	O: 82.9 (95% CI: 64.9–100%) after 11.6 years (3 out of 26) N: 98.4% (95% CI: 95.3–100%) after 13.7 years (1 out of 63)
BMI: overweight > 40 kg/m^2^ (O) vs. normal weight (N) (log rank test *p* < 0.001)	O: 50.0 (95% CI: 1.0–99.0%) after 8.9 years (2 out of 4) N: 97.0% (95% CI: 92.8–100%) after 13.7 years (2 out of 85)

## Data Availability

The data presented in this study are available on request from the corresponding author. The data are not publicly available due to privacy issues.
